# Host–Pathogen Interactions: Insects vs. Fungi

**DOI:** 10.3390/jof7030162

**Published:** 2021-02-24

**Authors:** Ivan M. Dubovskiy

**Affiliations:** 1 Laboratory of Biological Plant Protection and Biotechnology, Novosibirsk State Agrarian University, 630039 Novosibirsk, Russia; dubovskiy2000@yahoo.com; 2 Siberian Federal Scientific Centre of Agro-BioTechnologies of the Russian Academy of Sciences, 630501 Krasnoobsk, Russia

Although many insects successfully live in dangerous environments exposed to diverse communities of microbes, they are often exploited and killed by specialist pathogens. In the process of co-evolution of insects and entomopathogenic microorganisms, they develop various adaptive systems that determine the sustainable existence of dynamic host–parasite interactions at both the organismic and population levels. Many different species of fungi are associated with insects. It should be noted that the diversity of fungi largely depends on the specific insect–fungus system. Thus, in the population of *Chilo suppressalis*, a serious pest of rice, in northern Iran *Beauveria bassiana*, *Akanthomyces lecanii, Akanthomyces muscarious*, *Metarhizium anisopliae*, *Hirsutella subulata*, and *Trichoderma* sp. persisted [1]. Lepidopteran forest-pest species *Ematurga atomaria*, *Cabera pusaria*, *Hypomecis punctinalis*, and *Orthosia gothica* were associated with members of Cordycipitaceae (*Akanthomyces muscarius* and *Cordyceps farinosa*) and fungi from families *Aspergillaceae*, *Nectriaceae*, *Mortierellaceae*, *Hypocreaceae*, etc. [2]. The host defences are designed to exclude the pathogen or mitigate the damage inflicted, while the pathogen counters with immune evasion and utilization of host resources. Transcriptome (RNAseq) analysis of immune response uncovers new abilities to study host–parasite systems. Study of cricket *Gryllus bimaculatus* transcriptome demonstrated high tissue-specific variety in inducing antifungal immune factors [3]. Entomopathogenic fungi (EPF) neutralize their immediate surroundings on the insect integument and benefit from the physiochemical properties of the cuticle and its compounds that exclude competing microbes. Interestingly, in some cases EPF have low virulence because plant phytochemicals can demonstrate antimicrobial activity on insects cuticle [4]. EPF interplay host defence with factors which regulate adhesion to the cuticle, cuticle degradation, stress management and toxins [5]. Thus *B. bassiana* express bassianolide and beauvericin toxins during infection of the bug *Triatoma infestans* [6] and proteases, chitinases and lipase in the presence of *C. suppressalis* cuticle probably to pass the insects defence faster [1]. It was found that EPF peroxisome-type and hexagonal crystal-like organelles (Woronin bodies) are required for appressorium differentiation and the topical infection of insect hosts [7]. Insects’ immune, detoxification, and antioxidant systems work synergistically to combat infections and mitigate stress. Some proteins demonstrate multifunctional properties, participating in metabolism, homeostasis, and pathogen recognition [8]. Besides, insect hormones such as juvenile hormone [9] and dopamine [10] have been suggested to be a potential mediator in the insects’ immunity against fungi.

The application of EPF in the field needs high-quality scientific support to establish the mechanisms of action and ways to improve fungal biological preparations [11,12]. There are some cases in which an insect’s microbiota [13] and nematodes [14] may influence the development of fungal infections. These facts could open new abilities for the development of a complex approach to plant biological protection.

I would like to thank all contributors to this Special Issue on “Host–Pathogen Interactions: Insects vs. Fungi” for their significant contributions to this Special Issue and for making it a highly successful and timely collection of studies. I am extremely happy that we received eleven reviews/original papers for publication. 
